# External breast prostheses after mastectomy: production and selection of a low-cost functional model to be performed in developing countries

**DOI:** 10.3389/fonc.2024.1440109

**Published:** 2024-08-07

**Authors:** René Aloisio da Costa Vieira, Matheus Sampaio Ibrahim, Lucas Guimarães de Souza Araujo, José de Assis e Souza Junior, Carla Simone Moreira de Freitas, Luiz Carlos Navarro de Oliveira

**Affiliations:** ^1^ Faculdade de Medicina de Muriaé/UNIFAMINAS, Muriaé, MG, Brazil; ^2^ Departamento de Cirurgia Oncológica, Divisão de Mastologia, Hospital de Câncer de Muriaé, Muriaé, MG, Brazil; ^3^ Programa de Pós-graduação em Oncologia, Hospital de Câncer de Barretos, Barretos, SP, Brazil; ^4^ Programa de Pós-graduação em Tocoginecologia, Faculdade de Medicina de Botucatu/UNESP, Botucatu, SP, Brazil; ^5^ Departamento de Oncologia Clínica, Hospital de Câncer de Muriaé, Muriaé, MG, Brazil

**Keywords:** breast neoplasms, external breast prosthesis, quality of life, mastectomy, developing countries

## Abstract

**Introduction:**

Breast cancer is one of the most common types of cancer affecting women. Despite advancements in early diagnosis, neoadjuvant therapy, and various treatment modalities, mastectomy remains a common procedure for many women. Although some women opt for reconstructive surgery (BR), many do not have the indication, desire, or opportunity to undergo this procedure.

**Methods:**

An easily manufactured, washable, lightweight, and inexpensive external breast prosthesis (EBP) model was developed specifically for the study. Participants were presented with five EBP models–one commercially available, three manufactured options, and one created for the study–and were asked to choose a prosthesis. We also evaluated the factors associated with non-adherence to EBP among women who had undergone mastectomy without BR. The chi-square test was used to assess adherence or non-adherence to EBP, while logistic regression was used to identify factors associated with non-adherence.

**Results:**

We introduced a low-cost, lightweight, washable EBP model. When participants were asked to choose between two prostheses, the silicone prosthesis was the first choice for 33.9% of the participants, while the prosthesis created for the study emerged as the second choice for 70.5%. Out of the 72 women assessed, 45.8% (33) opted not to use any of the EBP models. Our analysis revealed that age and BMI were significantly associated with non-use of EBP.

**Conclusion:**

Multiple barriers contribute to non-adherence to EBP, underscoring the need for interventions aimed at improving patient knowledge and adherence. This study introduces a lightweight, easily reproducible, and low-cost EBP model.

## Introduction

Breast cancer is the most common neoplasm affecting women, with high morbidity and mortality rates despite the use of mammography and multiple treatments (1, 2). Regrettably, a substantial number of patients, mainly in developing countries, present with advanced clinical stages. Even with the application of neoadjuvant therapy, mastectomy remains a necessary procedure, affecting cosmesis and quality of life (1, 2).

Currently, several surgical options are available for conservative treatment or breast reconstruction (3). However, owing to factors such as disease severity, poor physical condition (comorbidities), and personal preferences, many patients who are eligible for mastectomy are not candidates for breast reconstruction (4).

Although external breast prosthesis (EBP) models are commercially available in different sizes, along with bras designed to support them, their acquisition can be problematic for low-income patients in developing countries (5).

Some public hospitals or volunteer initiatives have addressed this issue by creating EBP models of different sizes using various materials (5); however, adoption is not always complete. Critical factors influencing adoption include the appearance of the prosthesis under clothing, comfort, ease of cleaning, size, weight, absence of skin irritation, durability, and cost (6). Various types of EBP models are available, and adherence to EBP is influenced by age, education level, and income, requiring the creation of affordable options (7). Although EBP models based on the shape of the contralateral breast is a simple solution, they are impractical on a large scale (5).

Therefore, this study aimed to create a new EBP, evaluate patient preferences and factors associated to the use of EBP.

## Materials and methods

This study was approved by the UNIFAMINAS Research Ethics Committee on May 2, 2023 (Certificate of Submission for Ethical Appraisal no. 68799223.2.0000.5105). This prospective cross-sectional study was conducted at the Muriaé Cancer Hospital (MCH), Muriaé, MG, Brazil.

### EBP production

**Figure 1:**
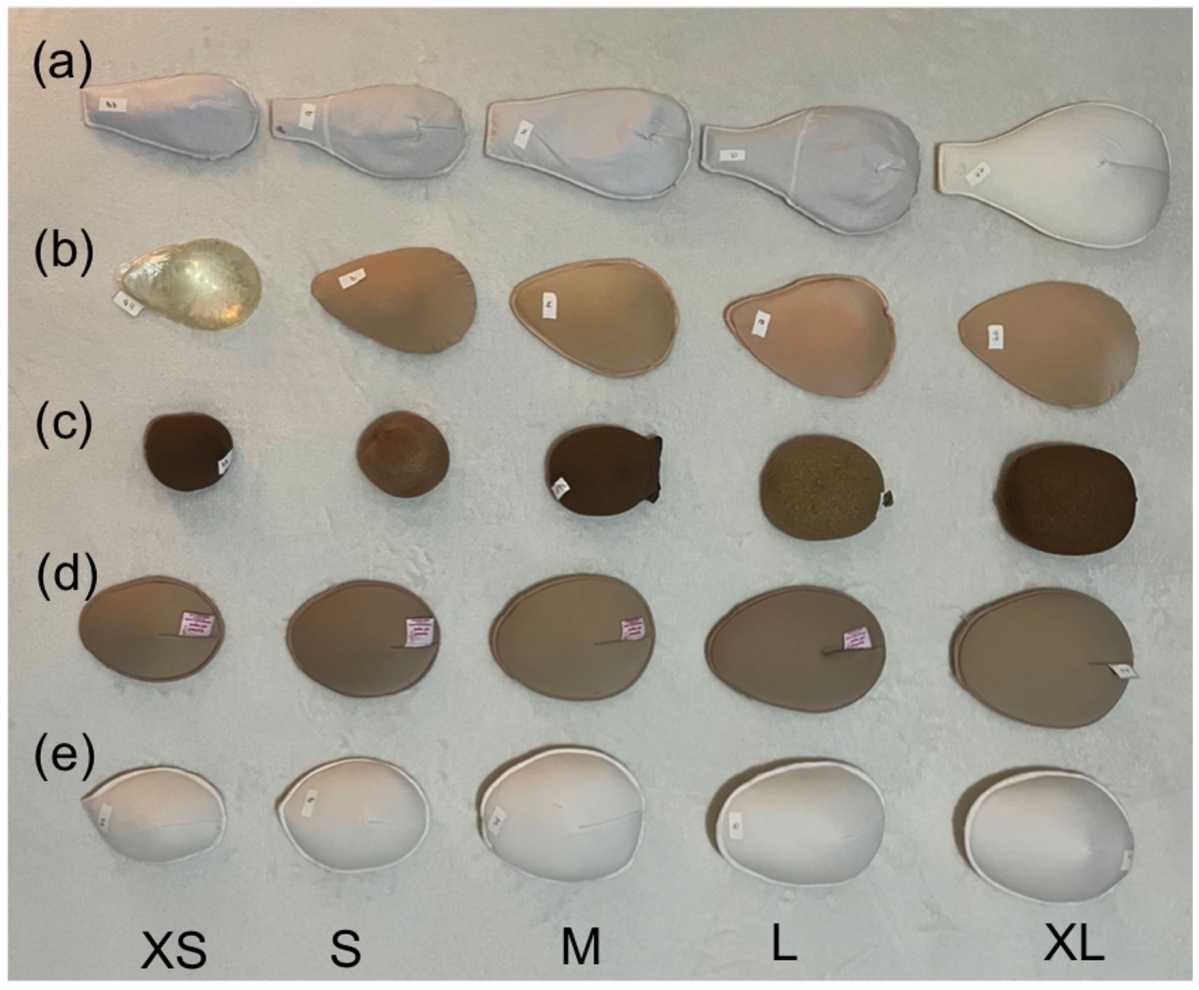
Prostheses used in this study.

The steps involved in the production of EBP models were as follows:

(1) Creation of a lightweight EBP mold.

(2) Adaptation of the mold to accommodate different breast sizes and projections.

(3) Production of a set of molds in different sizes.

(4) Weighing the final material.

(5) Comparison between commercial molds and molds made by volunteers used in our service and settings (**Figure 1**).

(6) Assessment of the cost associated with producing one EBP mold (**Table 1**).

**Table 1:** Characteristics of external breast prosthesis used in the study.

	Characteristics	Model 1 Cotton	Model 2 Silicone	Model 3 Birdseed	Model 4 Polypropylene	Model 5 Fiber
**Weight (grams)**	XS (PP)	58	123	137	91	19
	S (P)	72	304	204	150	24
	M	132	346	249	221	37
	L (G)	215	372	393	270	43
	XL (GG)	363	504	590	361	48
**Material**	Fabric	Cotton	Silicone/fabric	Nylon	Laminated foam	Laminated foam
	Lining	Granulated polypropylene	Silicone/fabric	Birdseed	Granulated polypropylene	Silicone fiber
**Cost (BRL)**	**-**	20	200	10	21	20
**Cost (USD)**		4	50	2	4.2	4
**Potential** **disadvantage**	–	Weight, Cotton	Weight High cost	Weight, Not washable	Weight	Study model

Currency rate: 1 USD = 5 BRL; Prosthesis size: US, XS-S-M-L-XL; BR, PP-P-M-G-GG.

The new EBP was developed and produced in collaboration with a clothing manufacturer located in Muriaé, MG, Brazil. The prosthesis was crafted from laminated foam filled with silicone fibers to ensure a lightweight and washable design.

In addition, commercially available silicone molds of various sizes were purchased, and three types of EBP were obtained from volunteers affiliated with oncology services (**Figure 1**). The prostheses were compared based on their materials, costs, and weights (**Table 1**). The cost analysis included an examination of the material and unit production costs in Muriaé, MG, Brazil.

### Participant assessment

The inclusion criteria were patients who underwent unilateral mastectomy without reconstruction and those who were undergoing post-breast cancer follow-up with no evidence of recurrence. They were recruited from either the Breast Surgical Oncology or Clinical Oncology outpatient clinic, where they provided informed consent before participating.

Participants were presented with the EBP models and completed a structured 10-minute questionnaire related to EBP usage, their preferred type of prosthesis, and the factors influencing their choice and maintenance of the prosthesis. Additionally, potential factors associated with EBP use, including time since surgery, age, education level, prosthesis size, clinical stage, and previous and current body mass indices (BMI) were assessed. Participants were shown the EBP models and asked to test them. Subsequently, they were asked about the two types of EBP they preferred and the factors potentially associated with their choice. Clinical data were retrieved from medical records.

We evaluated factors associated with the use of EBP. For this reason, we excluded patients who had undergone mastectomy less than 2 months before the study. The chi-square test was used to assess these factors, with Fisher’s test used when more than 20% of the cells had expected counts of less than 5. Univariate and multivariate logistic regression analyses were used to assess the factors associated with non-adherence to EBP. In the multivariate analysis, variables with a p-value <0.10 were considered for analysis, using a forward conditional model. Two models were assessed: Model 1 included participants with complete data (n=63), and Model 2 included participants with incomplete data for some variables (n=46). IBM SPSS® for Mac® version 20 was used for statistical analysis.

## Results

### EBP production

Our designed EBP models are characterized by lightweight and cost-effective construction, ensuring seamless integration with women’s clothing. They are made from an anti-allergic material (laminated foam) and feature washable properties (silicone fiber). A mold for each size (extra-small (XS), small (S), medium (M), large (L), and extra-large (XL)) was made by a seamstress using an A4 size (21 x 29.7 cm). These molds were developed to mitigate the potential disadvantages of EBPs and facilitate future EBP production in developing countries (see **Supplementary Material**). **Table 1**; **Figure 1** present the main aspects of the EBPs evaluated in this study. EBPs were developed to address the disadvantages of other models (**Table 1**), offering a replicable solution for low-income women receiving treatment within the public health system.

### Participant assessment

Among the 72 participants (**Supplementary Table 1**), the majority were aged 40–69 years (73.6 years on average). A significant proportion of participants were classified as overweight or obese (59.7%). Additionally, the majority had undergone mastectomy more than 12 months prior to the study (62.5%), had completed elementary or high school education (92.9%), and were diagnosed at an advanced clinical stage (85.1%). The mean time since mastectomy was 41 months, ranging from 1 to 215 months. Missing information was observed in some variables, as some patients underwent mastectomy at another hospital where incomplete information’s was recorded. These patients are now in follow-up care at our hospital.

Approximately 45.8% (n = 33) of the participants did not use EBP. The analysis of factors associated with non-adherence to EBP (n = 63) showed no association with time since surgery, age, education, EBP size, or clinical stage. However, age and current and previous BMI values were identified as variables associated with the absence of EBP use (**Supplementary Table 2**). Logistic regression (**Supplementary Table 3**), including the entire sample, showed that age was the primary factor associated with non-adherence to EBP, with patients aged ≥70 years at higher risk [odds ratio (OR) = 4.08] of non-adherence to prosthesis use. Additionally, the comprehensive BMI data revealed an association between previous BMI and the decision not to use a prosthesis, particularly noticeable among underweight participants (OR = 9.20).

Participants were asked to choose between two prosthesis options, with the silicone prosthesis being preferred by 33.9% and the prosthesis created specifically for the study selected by 70.5% as the second option. The factors considered most important for a prosthesis by the entire sample included weight (41.7%), shape (29.2%), comfort (15.3%), and ease of cleaning (12.5%).

## Discussion

The number of mastectomies without reconstruction is decreasing globally, influenced by multiple factors: (1) an increase in the therapeutic arsenal, improving response rates after neoadjuvant treatment; (2) the adoption of resection for residual disease after neoadjuvant therapy, allowing conservative treatment in patient’s initially eligible for mastectomy; (3) increase utilization of oncoplastic techniques associated with extreme oncoplasty; and (4) advancements in materials and surgical techniques, leading to immediate breast reconstruction with prostheses. Delayed breast reconstruction remains a viable option for patients who initially undergo mastectomy without immediate reconstruction because of neoplasia-related factors. These criteria often vary across institutions, leading to differences in breast reconstruction rates and techniques across both regionally and within the same country (8, 9). Global disparities in access to breast reconstruction surgeries persist. For instance, in India, less than 1% of patients undergo breast reconstruction (10), whereas, Brazil (11) and developed countries have experienced increased rates of immediate breast reconstruction (12), even among populations with racial disparities (10).

The use of EBP remains a significant issue in breast cancer, especially in developing countries where the high prevalence of locally advanced breast carcinoma contributes to elevated mastectomy rates (7). Reconstruction options may be restricted by insufficient skin coverage, which arises in cases of an unsatisfactory response to neoadjuvant chemotherapy, inflammatory carcinoma or hygienic mastectomy. Other situations are comorbidities, cultural aspects, race, individual income, or the patient preference for non-reconstruction (13). However, challenges persist owing to the limitations associated in the availability of reconstruction team (plastic, oncoplastic surgeon) in the context of public health system, and the costs of silicone prostheses, which represent a barrier to reconstruction for these populations (11).

Breast surgeons from our hospital (RACV and LCNO) evaluate reconstruction options based on the final tumor size after neoadjuvant therapy. In addition, they advocate for regular use of immediate breast reconstruction (IBR) with prostheses, resulting in a decline in the number of patients opting for mastectomy without reconstruction. Furthermore, patients who underwent mastectomy without IBR did not experience disease recurrence one year after treatment cessation, maintained a good clinical condition, and expressed a desire for reconstruction were eligible for delayed reconstruction using flaps. In cases of appropriate local conditions, the indications for early or delayed reconstruction are collaboratively discussed with the patient, who decides whether to proceed with reconstruction. The two surgeons perform breast reconstruction based on the indications and desires of the patient. These considerations may have an impact on the study results owing to the potential for biased selection among patients who underwent mastectomy without reconstruction. One study evaluating mastectomized patients reported increased satisfaction over time (14). In these scenarios, dissatisfied patients may opt for reconstruction during an extended follow-up period, potentially introducing selection bias.

Patients who undergo breast reconstruction generally experience a better quality of life compared to patients who do not (15). Additionally, the use of EBP models has been associated with better quality of life, as reported in some studies (15–17). However, some studies with a relatively small number of patients have reported no significant differences in quality of life comparing users and non-users of EBPs in unpaired case-control studies (18). Therefore, further studies on this topic are required. In Brazil, patients generally have access to EBP models, and the decision to use them is at their discretion. EBP usage can vary from occasional to daily (6), influenced by factors such as prosthesis size and weight (19). In this study, EBP usage was dichotomized based on the responses to the questionnaire, with negative responses indicating irregular use of the prosthesis. Several factors contribute to nonadherence to EBP, which may be intrinsic to the patient or related to the prosthesis. Patient-related factors included age, breast size, education level, indication for reconstruction (such as advanced stage), time since initial surgery, income, and self-esteem. Availability-related factors include the availability of materials, costs (prosthesis and accessories), limitations in replacing the prosthesis, and inadequacies in available models. Prosthesis-related factors include weight, material, size, ease of cleaning, and possibility of replacement (14). A study conducted in India reported that 40% of mastectomy patients used EBP, which was associated with a higher education level, younger age, and urban location (7). In our study, 54.2% of the participants used EBP, with non-use associated with older age (>70 years) and lower previous and current BMI values.

A previous study conducted in Singapore compared a commercial prosthesis (90% cotton and 10% elastane) with a manually crafted prosthesis by volunteers (100% cotton) and found superior results with the handmade EBP. However, the study lacked details regarding the content and weight of prostheses across different sizes (20). In Brazil, silicone is the most commonly used commercial EBP material. This study analyzed different models (**Figure 1**; **Table 1**) to explore aspects such as lining material, skin-contact material, size variations, and general characteristics. This detailed analysis facilitates comparisons with future studies and enhances our understanding of patient preferences.

Patients undergoing mastectomy without reconstruction often receive limited information about EBP (14), as the primary focus of the surgical team is on tumor treatment. In our study, many patients who did not use prostheses reported a lack of knowledge. This underscores the necessity for educational initiatives conducted by a multidisciplinary team at the initiation of treatment and throughout follow-up (21).

The use of EBP may lead to secondary postural changes; however, this aspect remains controversial (22). A study that analyzed silicone prostheses examined the relationship between prosthesis weight and shoulder pressure, reported a positive relationship, and emphasized the importance of low-weight prostheses in physical activities (23). However, another prospective controlled study reported contradictory findings (24).

In this study, we assessed the EBP-related factors that may potentially affect discomfort (6), focusing on weight, lining material, filling material, cleaning instructions, durability, and affordability of replacement. We analyzed several commercially available models and developed a washable, low-cost, non-allergenic prosthesis, in addition to providing a mold to patients (**Supplementary Material**), which forms the basis of this publication. Furthermore, the model we created was well-accepted by the participants. The first EBP choice was silicone, which is heavier, more expensive, and washable. The literature discusses the relationship between breast weight loss and body dynamics, which may have influenced the initial assessment (22, 23). Additionally, studies have highlighted significant costs associated with the acquisition of silicone EBP (25), posing a limitation for low-income populations. The second type of EBP was specifically created for this study. To our knowledge, no study has previously reported the provision of a model directly to patients, which is the primary novelty of this publication. In this study, molds were provided so that participants could have their EBPs crafted by local seamstresses. The selected material, laminated foam, offers washability and hypoallergenic properties, and the use of silicone fibers is associated with a lower weight (**Table 1**).

The limitations of this study include potential selection bias arising from the specific characteristics of the service, thereby limiting the generalizability of the results. Additionally, one limitation of this study is its small sample size, which we considered as indicative of a pilot study, providing information for future studies. We evaluated 72 patients and identified potential factors associated with the non-use of EBPs. Another limitation is the transverse design of the study. The study aimed to report on a new prosthesis and evaluate its potential choice within a limited time. The findings were solely based on the patients’ immediate choices, limited time, and medium-term prosthesis analysis, which could potentially alter the observed results. We were able to present the EBPs to the patients, but long-term evaluation and provision to patients were not feasible owing to lack of funding and Study planning. Therefore, future studies focusing on patient choice over time and quality of life in this population are warranted.

For future perspectives, it is important to evaluate the quality of life among patients who do not use EBP because of their unique characteristics. We anticipate widespread acceptance of the EBP models developed in this study. These models will serve as valuable resources to facilitate prosthesis acquisition and usage among patients with limited resources to acquire commercial EBP.

## Conclusion

This study introduced a lightweight, easily reproducible, low-cost, and effective EBP model. Our analysis revealed that age and BMI were significantly associated with non-use of EBP within the sample. These findings underscore the presence of multiple barriers related to non-adherence to EBP. Therefore, there is a critical need to improve patient education and promote adherence to EBP.

## Data availability statement

The raw data supporting the conclusions of this article will be made available by the authors, without undue reservation.

## Ethics statement

This study was approved by the UNIFAMINAS Research Ethics Committee on May 2, 2023 (Certificate of Submission for Ethical Appraisal no. 68799223.2.0000.5105). This prospective cross-sectional study was conducted at the Muriaé Cancer Hospital (MCH), Muriaé, MG, Brazil. The studies were conducted in accordance with the local legislation and institutional requirements. The participants provided their written informed consent to participate in this study.

## Author contributions

RV: Conceptualization, Data curation, Formal analysis, Funding acquisition, Methodology, Project administration, Supervision, Visualization, Writing – original draft, Writing – review & editing. MI: Data curation, Investigation, Visualization, Writing – original draft, Writing – review & editing. Ld: Data curation, Investigation, Visualization, Writing – original draft, Writing – review & editing. JS: Conceptualization, Methodology, Visualization, Writing – review & editing. Cd: Investigation, Visualization, Writing – review & editing. Ld: Conceptualization, Methodology, Visualization, Writing – review & editing.
