# Supplementary material for: External breast prostheses after mastectomy: production and selection of a low-cost functional model to be performed in developing countries
Source: Front Oncol. 2024 Aug 7;14:1440109. doi: 10.3389/fonc.2024.1440109 (PMC11335509; doi:10.3389/fonc.2024.1440109)
Supplement: Supplementary file 1 [file DataSheet_1.pdf]

frontiers

Supplementary Material

External breast prostheses after mastectomy: production and selection of a low-cost functional model to be performed in developing countries.

Legend

S. Material		Pages
S. Figures 1-2	Size PP/XS	1-2
S. Figures 3-4	Size P/S	3-4
S. Figures 5-6	Size M	5-6
S. Figures 7-8	Size G/L	7-8
S. Figures 9-10	Size GG/XL	9-10
S. Table 1	Characteristics of the study variables..................................	11
S. Table 2	Factors related to non-use of breast prosthesis.................	12
S. Table 3	Logistic regression of variables associated with non-use of breast prosthesis...	13

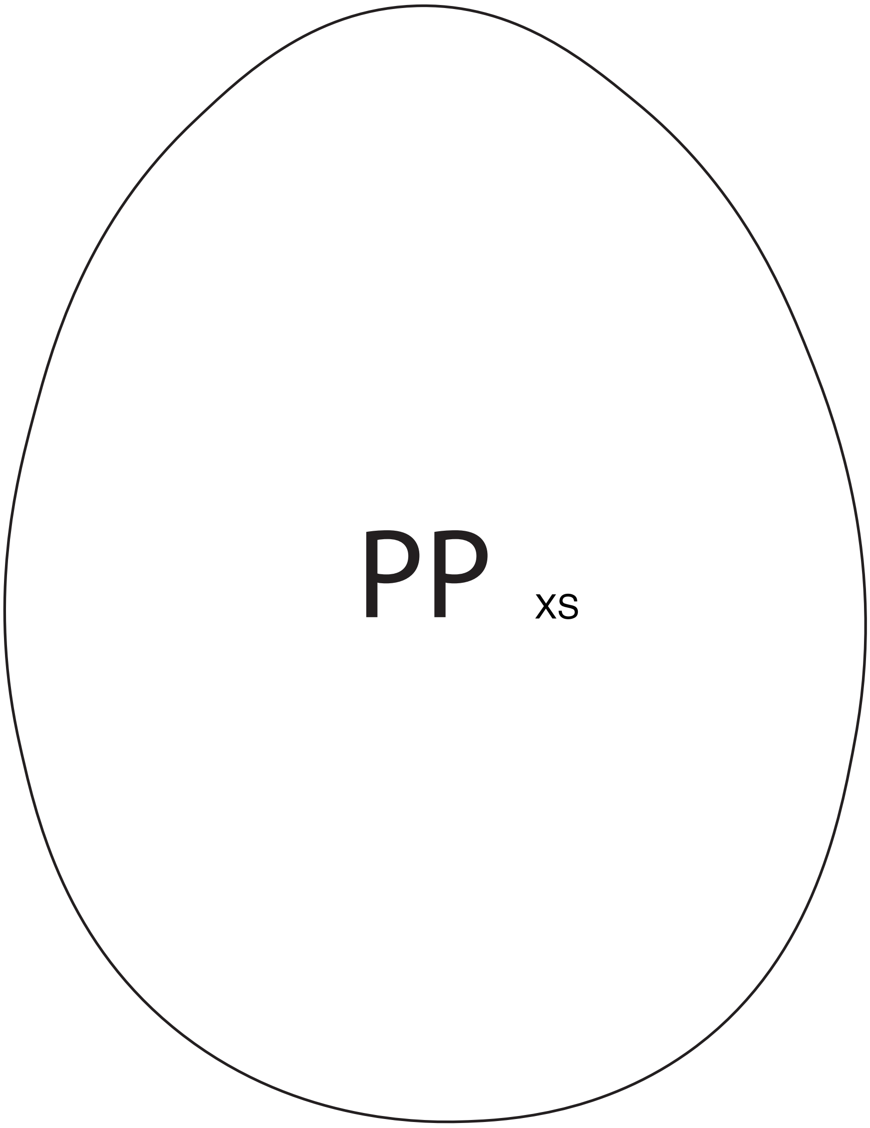
PP xs

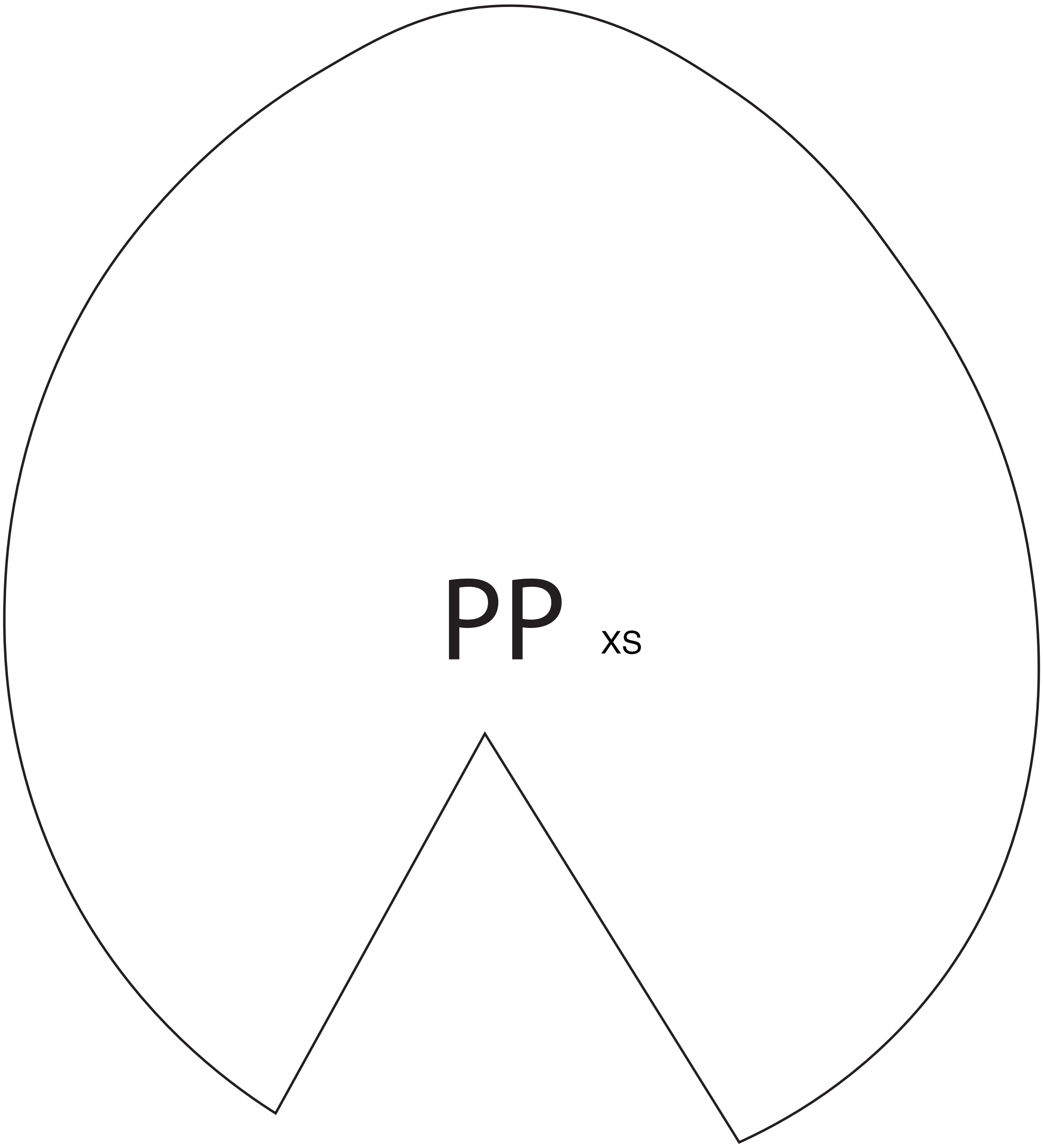
PP xs

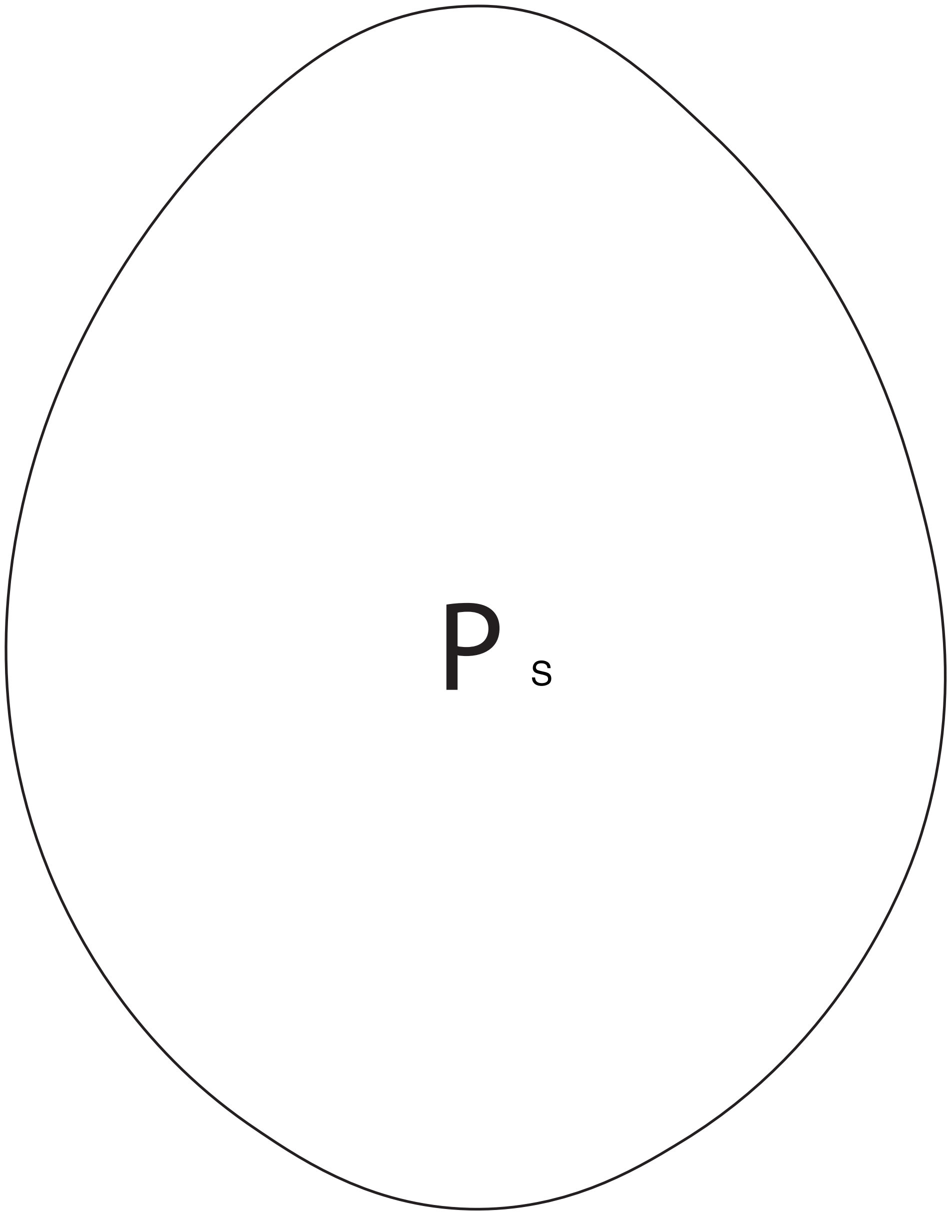
P s

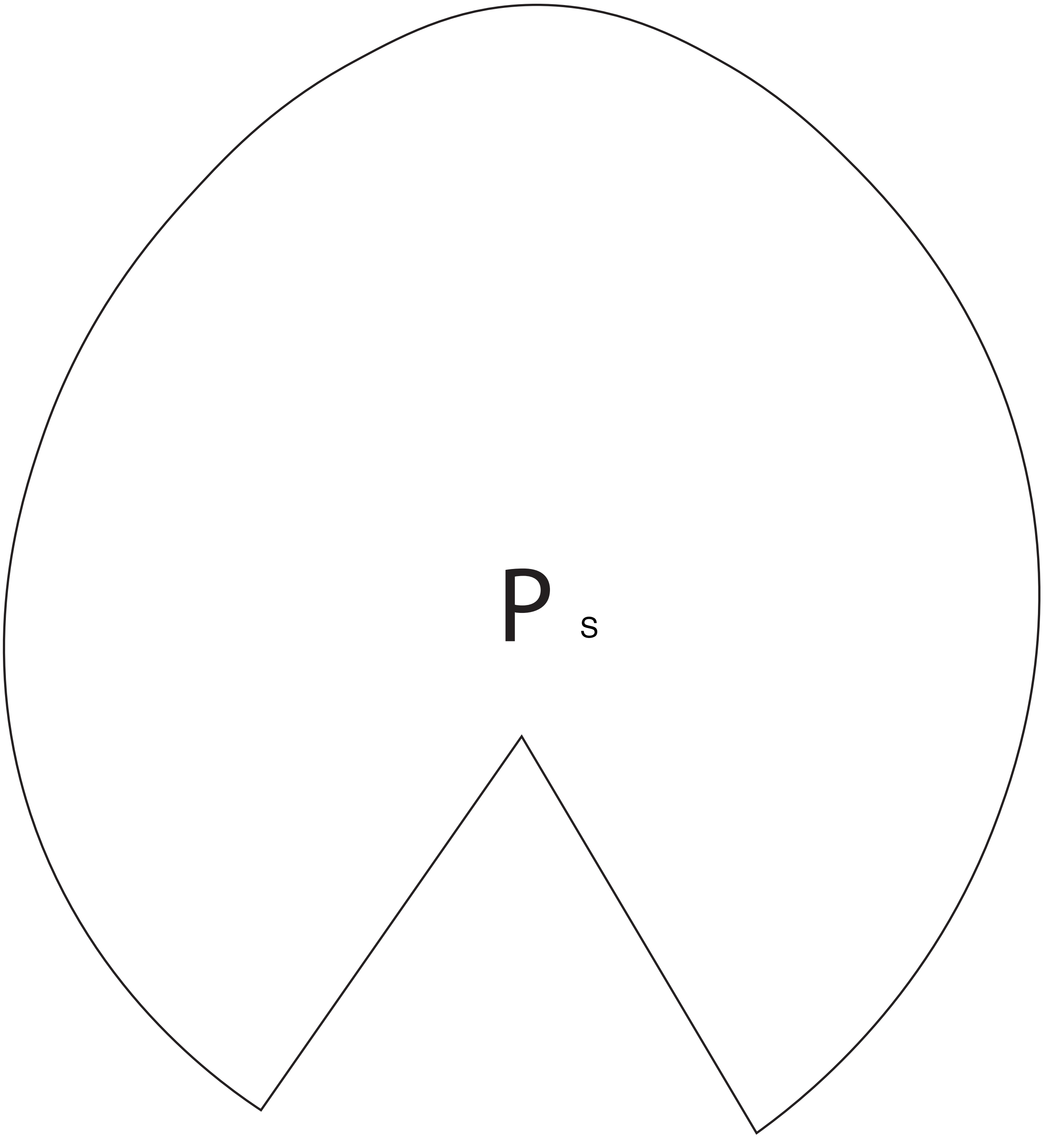
P s

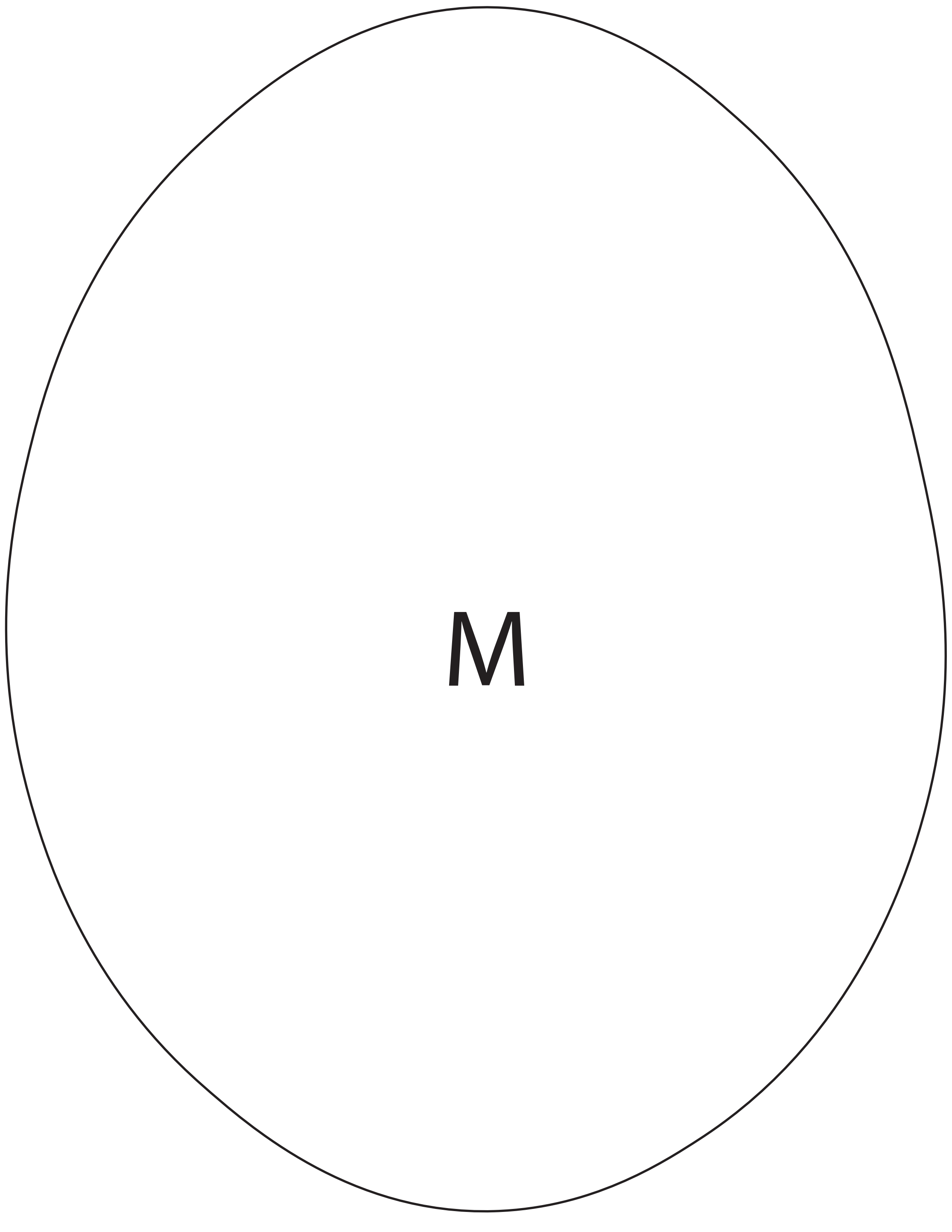
M

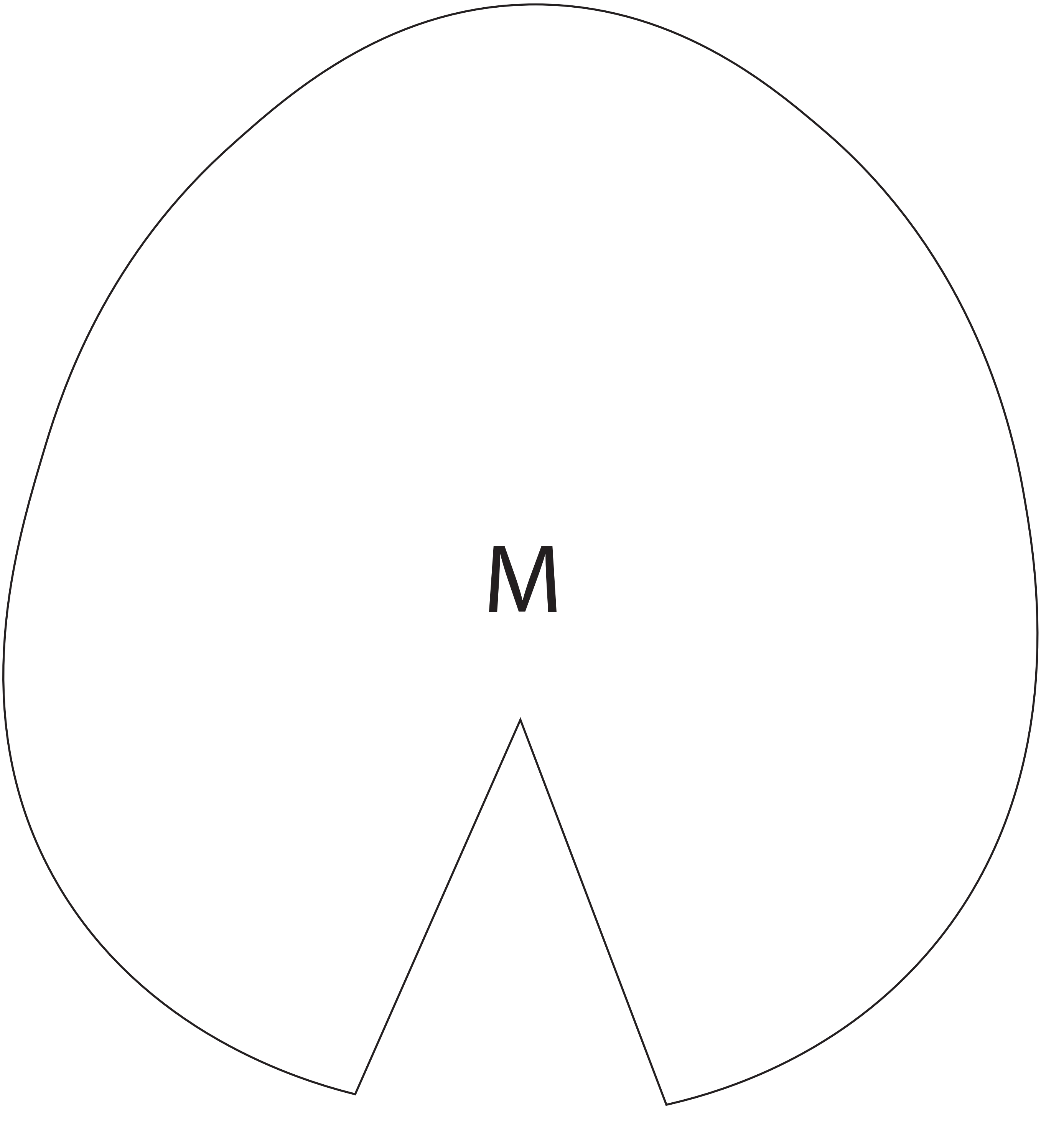
M

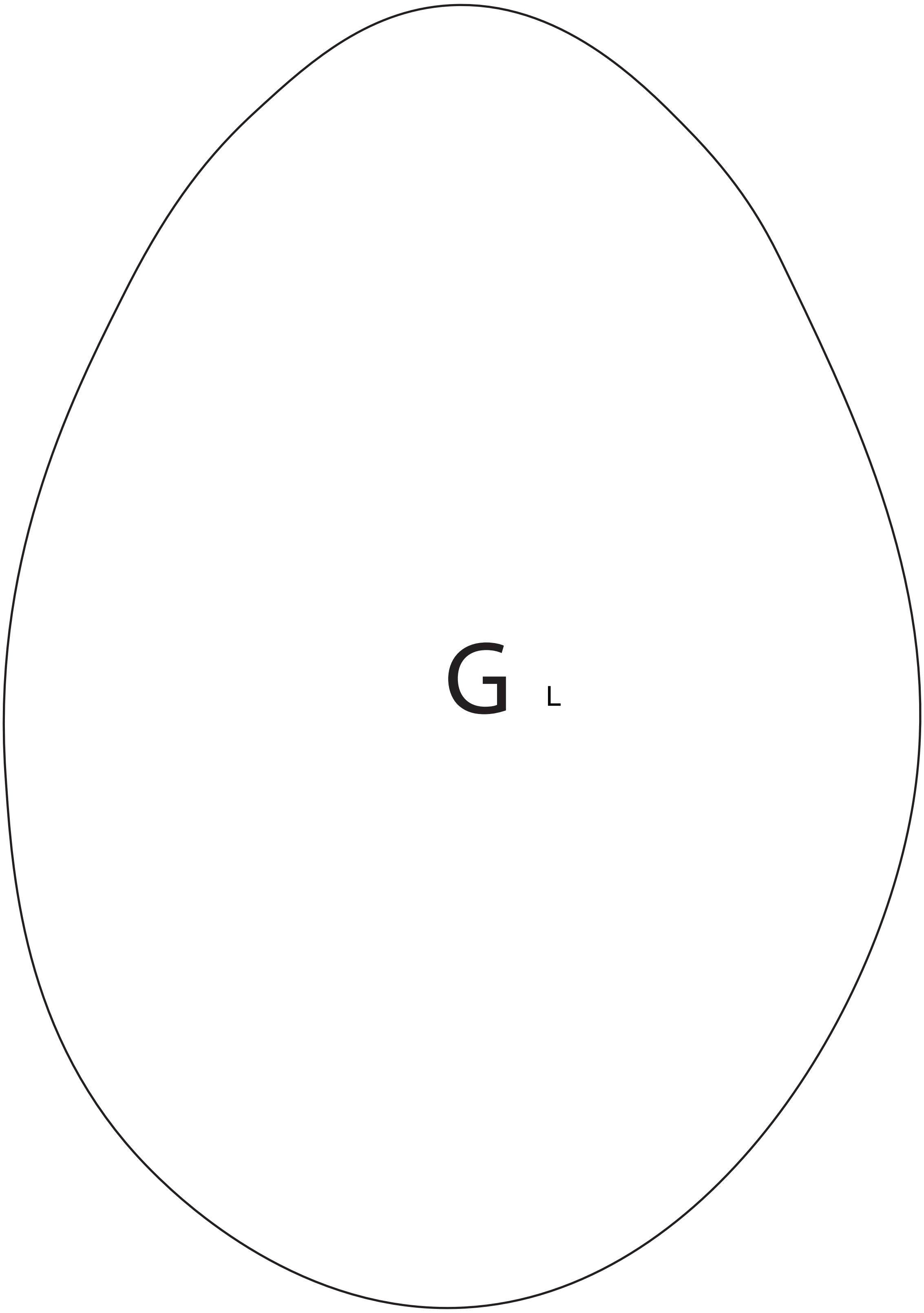
G L

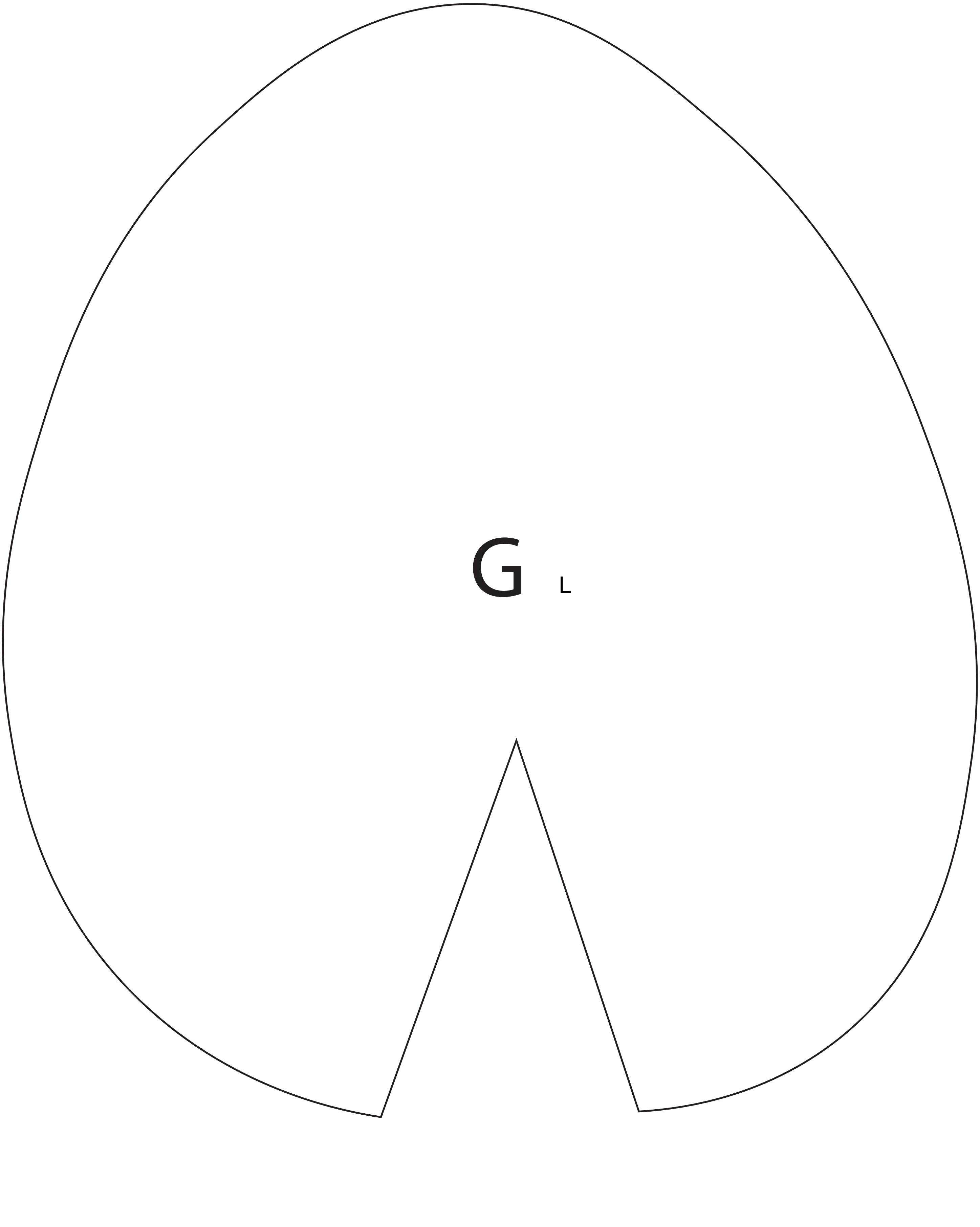
G
L

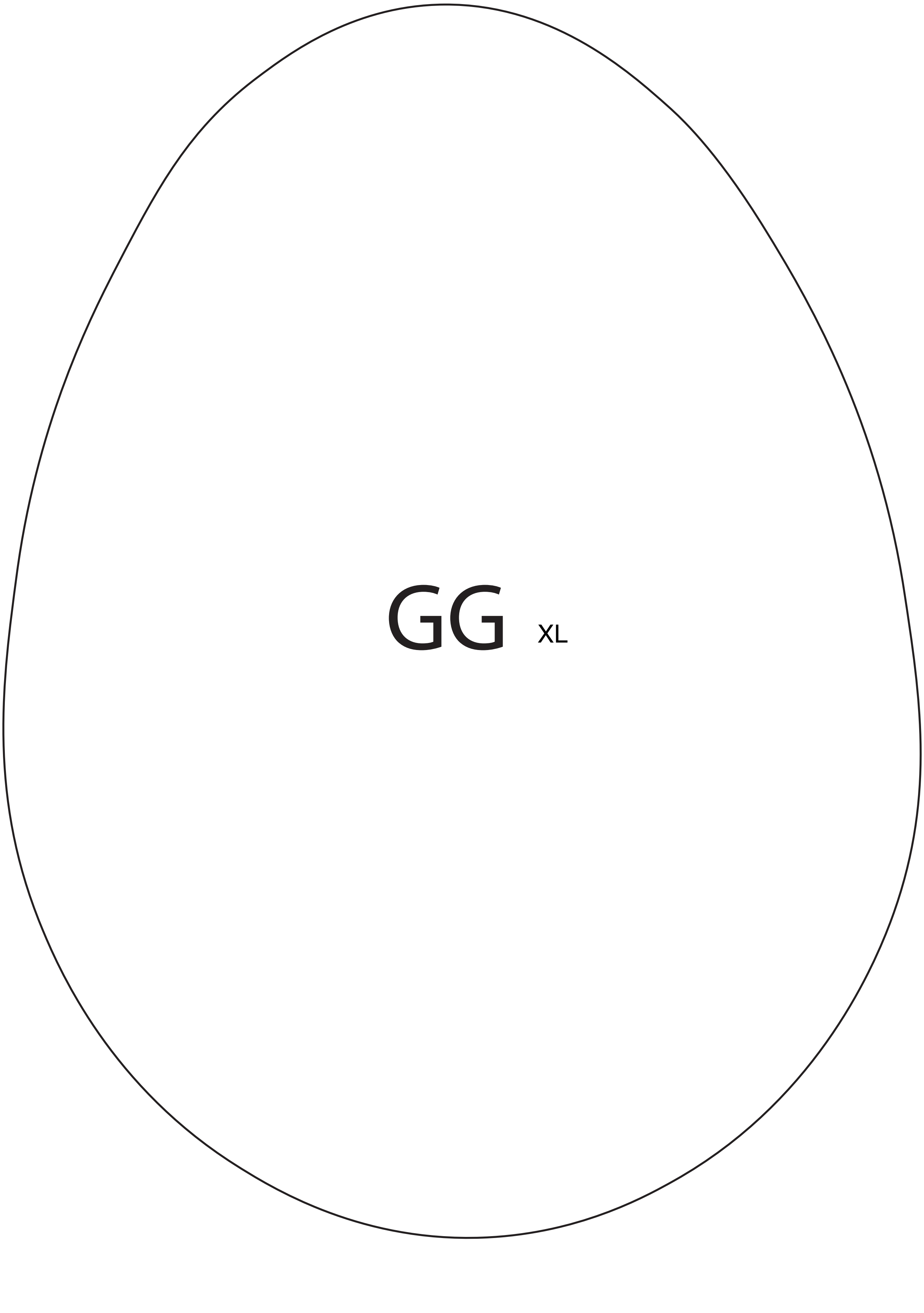
GG XL

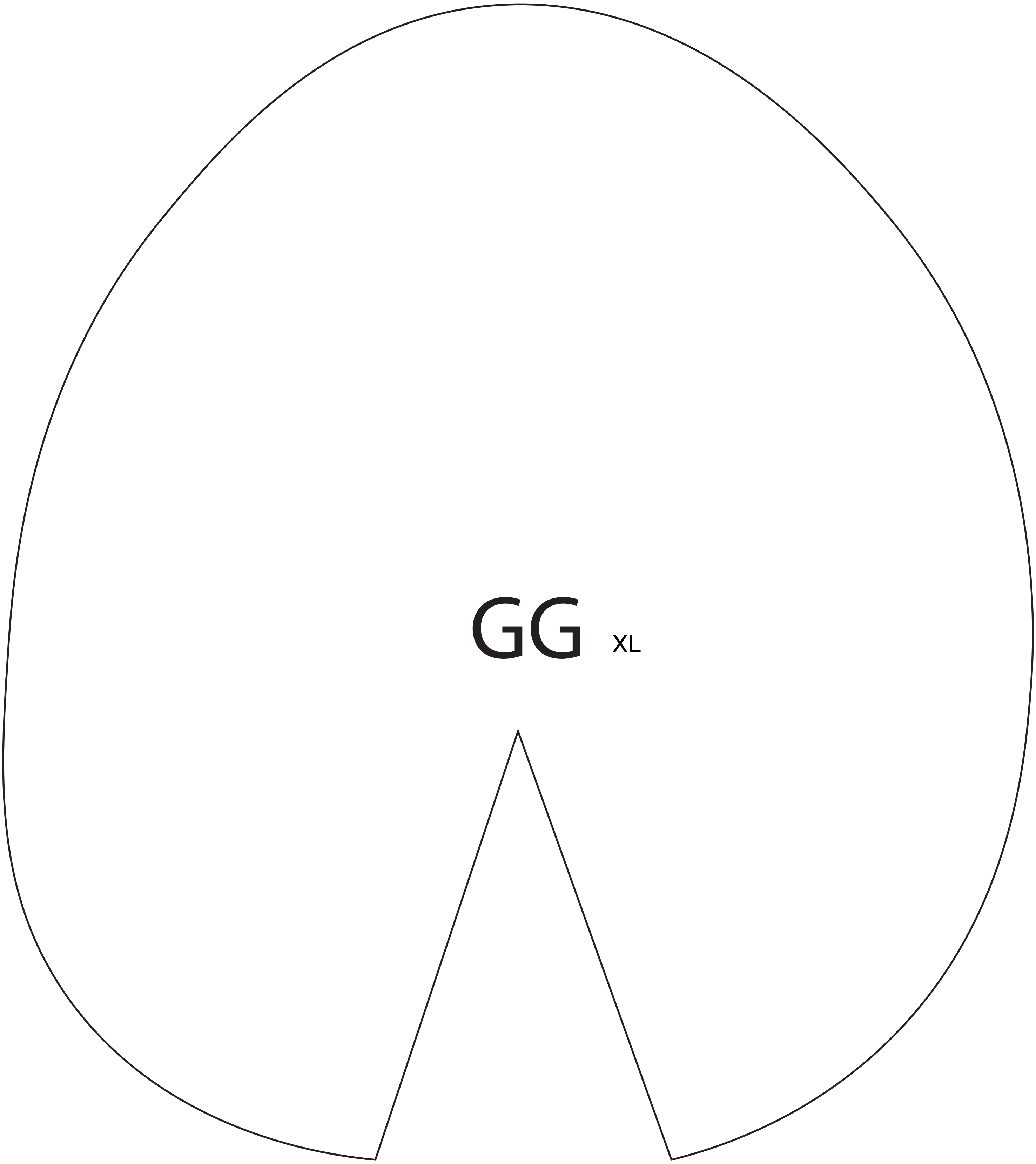
GG XL

Supplementary Table 1. Characteristics of the study variables.

Variable	Category	N patients	Percentage
**Patients**			
Age range	< 40	3	4.2
	40-69	53	73.6
	> 70	16	22.2
Current BMI	Low weight	5	6.9
	Normal	24	33.3
	Overweight	26	36.1
	Obesity	17	23.6
Time since mastectomy	Up to 1 month	9	12.5
	1-12	18	25.0
	>12	45	62.5
Education *	1º degree	27	38.0
	2º degree	39	54.9
	3º degree	5	7.0
Clinical stage*	CE I	10	14.9
	CE II	19	28.4
	CE III	38	56.7
**Breast prosthesis**			
Breast size	XS	12	16.7
	S	19	26.4
	M	25	34.7
	L	13	18.1
	XL	3	4.2
Use of Prosthesis	Yes	39	54.2
	No	33	45.8
1st choice	Silicone (2)	20	27.8
	Polypropylene (4)	16	22.2
	Lining (5)	10	13.9
	Cotton (1)	9	12.5
	Birdseed (3)	4	5.6
	No response	13	18.1
2nd choice	Lining (5)	43	59.7
	Polypropylene (4)	10	13.9
	Silicone (2)	4	5.6
	Birdseed (3)	2	2.8
	Cotton (1)	2	2.8
	No response	11	15.3

* Variable with missing data

Supplementary Table 2. Factors related to non-use of breast prosthesis.

Variable	Category	Present	Absent	Total	p
Age range_2	< 70	34 (69.4)	15 (30.6)	49	0.03
	≥70	5 (37.5)	9 (64.3)	14	
Age range_3	<40	2 (100)	0 (0)	2	0.05
	40-69	32 (68.1)	15 (31.9)	47	
	> 70	5 (35.7)	9 (64.3)		
Education	1º degree	13 (54.2)	11 (45.8)	24	0.52
	2º degree	23 (67.6)	11 (32.4)	34	
	3º degree	2 (50.0)	2 (50.0)	4	
Current BMI	Low weight	1 (25.0)	3 (75.0)	4	0.04
	Normal	10 (47.6)	11 (52.4)	21	
	Overweight/Obesity	28 (73.7)	20 (26.3)	38	
Previous BMI*	Low weight	1 (33.3)	2 (66.7)	3	0.03
	Normal	7 (46.7)	8 (53.3)	15	
	Overweight/Obesity	23 (83.1)	5 (17.9)	28	
Size	XS-S	15 (51.7)	14 (48.3)	29	0.30
	M	15 (71.4)	6 (28.6)	21	
	L-XL	9 (69.2)	4 (30.8)	13	
Prosthesis time	1-12 months	10 (55.6)	8 (44.4)	18	0.57
	> 12 months	29 (64.4)	16 (36.6)	45	
Clinical Stage*	CS I	3 (37.5)	5 (62.5)	8	0.30
	CS II	11 (64.7)	6 (35.3)	17	
	CS III	22 (66.7)	11 (33.3)	33	
Total	-	-	-	63	

* Variable with missing data

Supplementary Table 3. Logistic regression of variables associated with non-use of breast prosthesis.

Variable	Category	Risk ratio	IC	p variable	p total
Univariate					
Age range	< 70	reference	-	-	0.028
	≥70	4.08	1.16-14.25	-	
Previous BMI*	Overweight/obesity	reference	-	-	0.036
	Normal	5.26	1.29-21.35	0.020	
	Low weight	9.20	0.69-122.38	0.093	
Current BMI	Overweight/obesity	reference	-	-	0.054
	Normal	3.08	1.01-9.44	0.049	
	Low weight	8.40	0.78-90.36	0.079	
Multivariate[1]					
Age range	< 70	reference	-	-	0.028
	≥70	4.08	1.16-14.25	-	
Multivariate[2]					
Previous BMI*	Overweight/obesity	reference			0.036
	Normal	5.26	1.29-21.35	0.02	
	Low weight	9.20	0.69-122.38	0.09	

Multivariate analysis: (1) Model 1: patients with complete data: current BMI, age range
(2) Model 2: excluding variables with incomplete data; all model: current BMI, previous BMI, age range
